# “Does Physical Exercise Promote Health Benefits for Diabetic Patients during the COVID-19 Pandemic?”: A Systematic Review

**DOI:** 10.3390/sports11100192

**Published:** 2023-10-03

**Authors:** Erivaldo de Souza, Daniela Meneses-Santos, Josué Cruz Santos, Felipe J. Aidar, Carla Roberta de Oliveira Carvalho, Jymmys Lopes dos Santos, Anderson Carlos Marçal

**Affiliations:** 1 Postgraduate Program of Physical Education, Universidade Federal de Sergipe, São Cristóvão 49100-000, SE, Brazil; proferisouza@gmail.com (E.d.S.); josue.cruz.santos@gmail.com (J.C.S.); fjaidar@gmail.com (F.J.A.); jymmys.lopes@gmail.com (J.L.d.S.); 2 Department of Morphology, Universidade Federal de Sergipe, São Cristóvão 49100-000, SE, Brazil; danyymeneses@yahoo.com.br; 3 Department of Physical Education, Universidade Federal de Sergipe, São Cristóvão 49100-000, SE, Brazil; 4 Department of Physiology and Biophysics, Institute of Biomedical Sciences, Universidade de São Paulo, São Paulo 13566-590, SP, Brazil; croc@icb.usp.br

**Keywords:** physical activity, physical exercise, type 1 diabetes mellitus, type 2 diabetes mellitus, glycemic control, quality of life, SARS-CoV 2, COVID-19

## Abstract

Patients affected by COVID-19 are prone to facing disorders in multiple systems and organs, which can lead to deleterious diseases; in addition, people with pre-existing diseases may be more prone to the worst outcomes, and the most vulnerable are patients with type 1 and type 2 diabetes mellitus. The aim of this systematic review was to evaluate the effects of physical activity and/or physical exercise prescribed to individuals with diabetes on the maintenance of plasma glucose and glycated hemoglobin during the COVID-19 pandemic. Studies were found by searching PubMed, SCOPUS, Embase, Web of Science, SciELO, LILACS, SportDiscus, Bireme/BVS and Google Scholar databases. The inclusion criteria were articles that addressed only patients with type 1 or type 2 diabetes (T1D and T2D) who had evaluated the level of physical activity or physical exercise and described the effects on plasma glucose and/or glycated hemoglobin in cross-sectional, retrospective, and observational studies, meeting the main criteria established by GRADE. The PICO and GRADE strategies were used to select and assess the methodological quality of studies. Two reviewers searched and selected the articles in databases independently and blindly, during which oppositions and disagreements about the inclusion of articles were discussed and resolved by a third reviewer. Evidence corroborates that levels of physical activity were reduced due to the lockdown, leading to increased body weight and worse glycemic control. On the other hand, individuals with diabetes mellitus (DM) (T1D and T2D) who maintained and/or increased levels of physical activity or physical exercise showed reduced plasma glucose and glycated hemoglobin (HbA1c) levels. Adequate levels of physical exercise and physical activity are beneficial for glucose and HbA1c control in diabetic patients (type 1 or type 2). In addition, maintaining adequate levels of physical activity can contribute to reducing health problems when these patients are infected with COVID-19.

## 1. Introduction

The COVID-19 pandemic, which began in 2019 in Wuhan, China, led to several measures of social isolation with the aim of reducing the rapid transmission and spread of the virus, causing until August 2023 around 769 million confirmed cases, including 6,955,141 deaths [1,2].

This is because Severe Acute Respiratory Syndrome 2 (SARS-CoV 2), also known as coronavirus (COVID-19), is capable of promoting deleterious effects on infected individuals; among the most common are acute respiratory distress syndrome (ARDS) (also known as “cytokine storm”), increased production of prothrombotic factors, as well as endothelial dysfunction and respiratory failure [2,3,4,5].

In addition to these multiple disorders caused by COVID-19, some authors have reported that patients with diabetes affected by COVID-19 have a high risk of serious infections and a greater risk of mortality [6,7]. Studies have suggested that 11–58% of all individuals affected by COVID-19 are diabetic, and of these, 8% die [7,8]. The risk of evolution with the need to stay in intensive care units (ICU) among patients with diabetes mellitus (DM) is 14.2% higher than that of patients without the disease [9].

In this context, regardless of type of diabetes (type 1 diabetes (T1DM) or type 2 diabetes (T2DM), when affected by COVID-19, patients may be more susceptible to the development of the most severe form of the disease and, as a consequence, contribute to the increase in the number of deaths in the world population [7,10].

These factors are also encouraged by the decrease in time spent commuting with physical, occupational, and leisure activities, as well as the increase in time spent in sedentary activities, such as watching television and using electronic devices, a situation that has intensified with isolation measures caused by the pandemic of the new coronavirus and is a possible contributor to the increase in overweight and obesity numbers worldwide [11].

In this sense, in addition to social distancing measures and the use of personal protective equipment, such as masks, measuring body temperature, and assessing circulating oxygen saturation (oximetry) [5,7], it is also important, especially for individuals with diabetes, to maintain body weight and plasma glucose concentrations close to those recommended by world organizations [12,13,14,15]. 

In order to mitigate the effects of DM, in addition to adopting healthy eating habits, regular physical activity (PA) and exercise are also recommended. A recent study points out that PA is directly associated with numerous health benefits at any age, and since lifestyles are moving towards more sedentary profiles, it is essential to think about strategies to promote healthier habits [16]. Studies that evaluate such benefits are extremely important since various results point to improved health from the practice of PA, such as the improvement of factors associated with inflammation, metabolism, glycemic status, and lipid profile in patients with T2DM [17].

Corroborating the aforementioned findings, a systematic review and meta-analysis including 26 randomized clinical trials with a total of 3300 T2DM participants found that aerobic exercise can improve waist circumference in people with T2DM [18]. Thus, it is well studied that aerobic training programs help in improving body composition, with weight loss, insulin sensitivity, a decrease in HbA1C levels and blood pressure, an increase in maximum oxygen uptake (VO2max), and decreased mortality rates [12,13,14,15,19,20].

Given that weight gain, progression to obesity, and associated diseases, especially DM, increase the risk of hospitalization, the need for an Intensive Care Unit (ICU), and death among patients with COVID-19, it is suggested that PA may be important to mitigate the worsening of metabolic and vascular-respiratory disorders caused by COVID-19 [21,22].

These guidelines are important since glucose within values considered as control during hospitalization was associated with low mortality, whereas for individuals with hyperglycemia, a high mortality rate was observed, indicating that individuals hospitalized for COVID-19 with T2DM with decompensated glucose had a high risk of death compared to others [21,22].

Small PA “doses” throughout the day to break sitting can attenuate postprandial glucose and insulin levels, particularly in individuals with insulin resistance [14], in which regular aerobic exercise improves glycemic control in adults with type 2 diabetes, with a shorter daily time of hyperglycemia and a reduction of 0.5–0.7% global glycemia (as measured by HbA1c) [14].

In general, for the control and prevention of DM (T1DM and T2DM), at least 150 min per week of moderate-intensity aerobic physical activity or 75 min per week of vigorous-intensity aerobic activity, or an equivalent combination of both, is recommended [13,14,18].

In this sense, the regular practice of physical activity seems to promote metabolic adjustments that contribute to a less severe clinical outcome of the disease in patients diagnosed with COVID-19 [8,9,12]. However, there is little information regarding the standardization of the practice of physical activity and exercise and its effects on metabolism in DM patients affected by COVID-19 [10,13]. Thus, the aim of this review is to evaluate the effects of physical activity and exercise on individuals with diabetes diagnosed with COVID-19.

## 2. Materials and Methods

### 2.1. Study Design, Sources, and Search Strategy

This is a systematic review study using the PICO [23] strategy for the elaboration of the problem question, recommended for database searches according to the Preferred Report Items methodology for systematic and staged reviews. analyzes (PRISMA), considered relevant for the construction of reviews [24].

This systematic review aimed to evaluate the effects of physical activity and/or physical exercise prescribed to individuals with diabetes on the maintenance of plasma glucose and glycated hemoglobin during the COVID-19 pandemic. For the construction of the question and objective problem, the PICO [23] (patient, intervention, control, and outcome) strategy was used, following the PRISMA methodology [24] (Preferred Reporting Items for Systematic Reviews and Meta-analyses). The protocol of this systematic review was registered on the PROSPERO platform (International Prospective Registry of Systematic Reviews) with the identification number [CRD42022365123] [25].

### 2.2. Search Strategies

**Figure 1:**
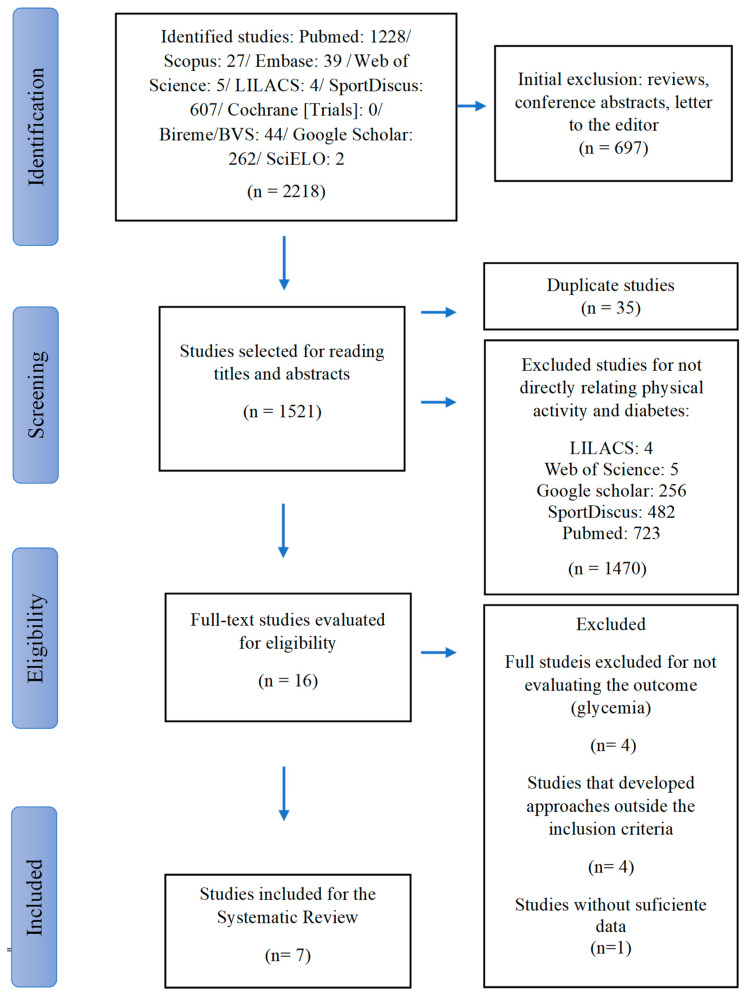
Systematic literature review flowchart.

Descriptors (as recommended by Descriptors in Health Sciences (DeCS) and Medical Subject Headings (MeSH)) were chosen and organized into three domains: (a) COVID-19: COVID-19 OR coronavirus OR 2019-ncov OR sars-cov-2 OR cov-19 OR “coronavirus”; (b) DIABETES: diabetes OR “diabetes mellitus” OR “diabetes type 2” OR “diabetes mellitus type 2” OR “diabetes 2” OR "diabetes 2" OR diabetic; (c) EXERCISE: exercise OR “physical activity” OR “physical exercise” OR fitness OR “exercise training” OR “aerobic exercise," as shown in Figure 1. Articles were searched in the following databases: PubMed, SCOPUS, Base, Web of Science, SciELO, LILACS, SportDiscus, Bireme/BVS and Google Scholar. The search period was during the months of September and October, in Portuguese or English, considering studies carried out from March 2020 to 3 March 2023.

### 2.3. Inclusion of Studies

During the first step of the study selection, 2218 results were found, distributed in 10 electronic databases (Figure 1). After removing duplicate studies, 1521 articles remained for further analysis of titles and abstracts. Subsequently, for the next step, 16 studies were submitted for complete evaluation, with nine studies being eliminated. Thus, seven potentially eligible studies remained, which were carefully evaluated for further qualitative analysis (Figure 1).

### 2.4. Inclusion and Exclusion Criteria

The adopted inclusion criteria were elaborated according to Amir-Behghadami and Janati (2020) [26] and Richardson et al. (1995) [27]: Participants: patients with diabetes (T1DM or T2DM); Intervention: participants who performed physical exercise programs and/or physical activity monitored within the context of COVID-19; Control group: a control group consisting of diabetic patients (T1DM or T2DM) who did not undergo intervention or practice regular physical activity; Results-measures: data and results that included blood glucose concentration and/or HbA1c from randomized clinical trials (RCT) or observational designs, quasi- or experimental studies, original studies, human model studies, and articles published in English.

Exclusion criteria: studies that did not involve intervention with physical exercise or checking the level of physical activity; studies outside the context of COVID-19; studies performed in patients without diabetes; studies that did not analyze blood glucose or HbA1c outcome; systematic review articles with or without meta-analysis; studies with non-detailed intervention methods; repeatedly published articles, abstracts, editorials, letters to the author, and articles in languages other than English.

### 2.5. Procedure

Three steps were carried out with the collaboration of two independent and blind members, through the Rayyan manager [28,29] for the analysis and selection of studies. In case of conflicts, an additional element was included to achieve a final decision on the inclusion or exclusion, as follows: The researcher (ES) carried out the initial searches and removed duplicate articles as well as works that would not be included according to the initial exclusion criteria (reviews, conference abstracts, letters to the editor). In addition, two researchers (ES and DM) independently and blindly evaluated selected titles and abstracts, excluding those not directly relating to physical activity and diabetes. After this initial investigation, once there were disagreements, a third researcher (AM) resolved conflicts regarding the previously chosen works.

### 2.6. Data Extraction

Three steps were carried out: Step 1: Analysis of the Title: When eligible, the abstract was read according to inclusion criteria. Step 2: A full reading of the article based on previously established criteria Step 3: Inclusion of eligible studies in this final step, characteristics of studies such as country, experimental design, configuration, author(s), date of publication, sample characteristics (population, age of participants, type of diabetes, plasma glucose levels, HbA1c, physical activity or exercise performed, insulin administration (when indicated)).

### 2.7. Assessment of the Quality of Individual Studies

The GRADE system was used to assess the quality of the evidence. Seven studies were included, from which the number of participants, the evaluation protocol, and the values available for blood glucose, HbA1c, body weight, BMI, and insulin use were extracted, relating them to the pathology under study.

In order to synthesize the quality of evidence, the GRADE system (Grading of Recommendations Assessment, Development, and Evaluation) [30] was used as a way of representing confidence in the information provided, which classifies the level of evidence and expresses the emphasis for adopting or rejecting a certain conduct in clinical trial reviews. This systematic review analyzed only observational studies with a high level of evidence.

The GRADE system has the following criteria to be considered: design and methodological limitations of included studies; inconsistency (homogeneity of studies); determining whether the study presents direct evidence; accuracy of results; and verifying whether the study exposes a publication bias by not including all studies about the research problem.

Based on these criteria, the level of evidence was classified according to the four levels proposed by the GRADE system: “High quality"—it is very unlikely that additional research will change the results presented by the systematic review; “moderate quality”—further studies are likely to have an important impact and may change the results presented by the systematic review; “low quality”—it is possible that other studies have an important impact and are likely to change the results presented by the systematic review; and “very low quality”—any estimate of results presented by the systematic review is very uncertain and requires the development of further studies [30].

## 3. Results

### 3.1. Characteristics of Eligible Studies and Population/Sample Details

Eligible studies involved analysis of the effects of physical activity and/or physical exercise in patients with diabetes affected by COVID-19 from articles published between the years 2020 and 2023, as follows: 01 from Saudi Arabia [31], 01 from Japan [32], 01 from the Netherlands [33], and 04 from Italy [34,35,36,37] (Table 1).

The total sample of patients from eligible groups was composed of 1287 individuals aged 8–67 years. Regarding the type of study, 02 were cross-sectional studies [31,32] 02 were retrospective studies [36,37]; and 03 were observational studies [33,35] (Table 1).

### 3.2. Characterization of DM Patients Affected by COVID-19: Type of DM, Plasma Glucose Concentration and Administration of Exogenous Insulin during the Pandemic

In eligible studies, patients were classified according to the different types of diabetes (Table 1). Notably, 05 involved T1DM patients [31,34,35,36,37]; 01 evaluated T2DM patients [32]; 01 evaluated T1DM and/or T2DM patients [33].

Of the variables evaluated in eligible and discussed studies, those most cited by the authors were: plasma glucose (mg/dL) and HbA1c concentration (mmol/mol; %), in the same study [31,34,36]; or individually: analyzing HBA1C (mmol/mol; %) [32,33,34]; and/or plasma glucose concentration [37] (Table 2).

Regarding the average insulin dose (U/kg/day) administered to patients, 05 detailed the concentration and form of administration [31,32,35,36,37]; 02 studies did not report this information [33,34] (Table 2).

### 3.3. Characterization of Studies Regarding Levels of Physical Activity, Plasma Glucose, Body Weight, and HbA1c in DM Patients during Restrictions of the COVID-19 Pandemic

In the study by Al Agha et al. (2021) [31], in T1DM patients, a decrease in the practice of PA in the pandemic period was identified. Furthermore, approximately 64.9% of participants were not satisfied with the levels of PA associated with negative changes in lifestyle and weight gain. Similar results were found in the study by Assolani et al. (2020) [34] in T1DM patients, where a decrease in the number of steps/day and in the minutes spent performing PA was evidenced.

In another study [37], in T1DM patients, the authors reported that 76% of participants maintained regular PA during the quarantine and that all participants had good glycemic control during the social restriction established during the pandemic period, with blood glucose remaining at appropriate levels for this pathology (between 70 and 180 mg/dL), as recommended by Kanaly et al. (2022) [14].

When analyzing T1DM patients of different age groups (children, adolescents, and adults), moderate PA was significantly lower in adolescents [35]. However, no significant difference was identified in the metabolic parameters of adolescents for HbA1c and plasma glucose variables from continuous glucose monitoring (CGM) derived from 20 days before confinement and 20 days during lockdown.

Among young adults, plasma glucose concentration was improved in participants who performed PA (IPAQ: moderate physical activity/day, Mets, and IPAQ: walking/day, Mets). Furthermore, patients with increased glucose variability were those who presented greater stress perception (25% of the total) [35].

T2DM patients showed a decrease in the frequency of exercise, an increase in the consumption of processed foods, and increased stress perception during the pandemic period [27]. In this study, stress perception stands out since there was an inversely proportional relationship between stress levels and the practice of physical exercise. When comparing variables that influenced metabolic parameters, an inversely proportional relationship was observed between the practice of PA and food intake, which was corroborated by the increase in body weight and HbA1c concentration in this population.

Similarly, the study by Ruissen et al. (2021) [33] showed that 45.7% of diabetic participants reported that during the pandemic period, the practice of physical exercise was reduced, which was associated with an increase in stress perception and body weight. In this study, high stress was identified in 34.1%, with no difference between T1DM and T2DM (33.6% vs. 35.1%, respectively) being associated with a change in HbA1c. In addition, the greater the difficulty in controlling blood glucose, the higher the stress level. Furthermore, all participants presented high anxiety levels, with no differences between T1DM and T2DM (27.5% vs. 26.9%, respectively).

In the study by Minuto et al. (2021) [36], T1DM patients showed a significant reduction in the practice of weekly PA during the pandemic period. However, patients who practiced intense physical activity increased the time it took to reach adequate glucose levels; therefore, within the mean variability established as acceptable, from 56.91 to 64.11%, according to other authors [14], This study suggested that adopting a healthier lifestyle is associated with maintaining PA and glycemic control.

### 3.4. Quality of Studies

In assessing methodological quality and risk of bias, three studies were considered of high quality (score ≥ 60%). The mean methodological quality of studies was 53.84%, as can be observed in Table 3. The main areas of methodological weakness found were: inter-rater blinding; intra-rater blinding; variation of the evaluation order; time period between measurements; and adequacy of the reference standard description.

In view of the above and the quality of the evidence when considering the characteristics of studies, mainly regarding the methodological rigor adapted to the context of restrictions in which they were developed, it is likely that other studies will have an important impact and change the results presented by the present systematic review. In addition, it should be highlighted that most studies included in this review met most of the main criteria established by GRADE [30].

## 4. Discussion

The aim of this systematic review was to evaluate the effects of physical activity and/or physical exercise prescribed to individuals with diabetes on the maintenance of plasma glucose and glycated hemoglobin during the COVID-19 pandemic, including seven cohort studies of cross-sectional, observational, and retrospective characteristics with a total of 1.287 participants.

Our findings showed that during the quarantine period, decreases in levels of physical exercise and physical activity were observed [31,33,34]. These effects must have contributed, at least in part, to the increase in body weight [32,33] and, consequently, in BMI [31] and the increase in plasma blood glucose associated with increased plasma HbA1c concentration [27].

Maintaining the practice of PA by individuals diagnosed with T1DM and/or T2DM is able to promote beneficial adjustments in the body to maintain blood glucose levels close to parameters recommended by different international associations [13,14,15]. This suggests that increasing the level of PA involving the regular practice of physical exercises (PE) in a guided way seems to be essential for maintaining the health of DM patients [13].

PA combined with a healthy lifestyle resulted in improved glycemic control (GC) [36,37]. In addition, intense PA groups showed better GC compared to the group without PA [36]. Furthermore, performing moderate PA results in lower T1DM concentrations with a longer time under recommended parameters [30]. During the lockdown period, an unexpected improvement in GC was found in T1DM patients [37].

Thus, plasma glucose concentrations in DM patients above levels recommended by international organizations can result in cardiometabolic risks, marked insulin resistance, dyslipidemia, and hypertension. These events lead to the development of endothelial damage that may contribute to deleterious cardiovascular myocardial events in diabetic patients with metabolic dysregulation [38,39].

### 4.1. Maintenance of Adequate Levels of Physical Activity for Glycemic Control and Maintenance of HbA1c Concentration in Diabetic Patients during the COVID-19 Pandemic

Several studies suggest that maintaining adequate levels of PA, associated with the practice of other healthy habits such as diet and adequate sleep, is efficient for maintaining GC and adequate HbA1c concentrations, consequently improving the quality of life of T1DM and/or T2DM patients [40,41].

It is known that the practice of PA promotes improvement in body composition, lipid profile, weight loss, vascular health, and homeostasis between plasma glucose and insulin concentration in T1DM and T2DM patients; in addition, these metabolic adjustments can contribute to the reduction of the risk of diseases, especially cardiovascular ones [41,42,43].

Thus, evidence suggests that diabetic patients, regardless of type, should be encouraged to decrease levels of sedentary behavior and increase levels of PA [41], since, in a beneficial way for T1DM and T2DM patients, in the post-PA and training period, there is an increase in insulin sensitivity, and these effects are in part due to the improvement in glucose uptake by dependent and independent pathways of this hormone [44,45,46]. Furthermore, it should be highlighted that the metabolic adjustments promoted by PA depend on physical exercise intensity and duration as well as on a decrease in sedentary behavior. When performed on a regular basis, PA is also capable of promoting improvements in insulin sensitivity and reductions in adipose tissue [47].

As a consequence, physical inactivity caused by social isolation directly and indirectly contributed to the worsening of the clinical condition of diabetic patients affected by COVID-19 [8,9,10]. 

In addition to the social isolation caused by the pandemic, there are still several factors that can contribute to non-adherence to the practice of PA by diabetic patients, such as the type of sport, the multimodality management of DM athletes, and adequate knowledge about type and exercise intensity [9].

In this review, it was found that part of the participants were not satisfied with their level of PA [31]; however, the dissatisfaction was not enough to encourage them to change their sedentary behavior [33]. Such evidence suggests the existence of a vicious cycle in the pandemic period caused by social isolation involving increased sedentary behavior, decreased levels of PA, and increased body weight and plasma glucose [8,9].

In patients diagnosed with DM, the maintenance of the practice of PA and/or physical training at adequate levels, properly guided by trained professionals, combined with the adoption of healthy eating habits, can contribute to the reduction and/or maintenance of body weight and the stability of metabolic parameters such as reduced plasma glucose and HbA1c control [12,13,14,15].

For diabetic individuals, HbA1c close to 7.5% (with variability < 7%), associated with blood glucose concentration close to 175 mg/dl (maintaining variability 36%), is essential for the prevention of micro- and macro-vascular complications [12,13,14,15].

The practice of PA plays an important role in maintaining HbA1c concentrations close to recommended values. It was verified that T1DM patients who were submitted to 30 min/week of moderate to vigorous aerobic exercise showed improvement in HbA1c concentrations, with the greatest effect observed at 100 min/week and above [48]. However, physical exercise above 100 min/week seems to be ineffective for increments in the reduction of HbA1c concentration.

In another similar study, results suggest that programs with more than 24 weeks of training consisting of at least 60 min/session of high-intensity concurrent exercise can be used as an auxiliary therapy for metabolic control in T1DM patients [49].

Similarly, Nair et al. (2022) [50] found that when PA practice consists of regular walks, it is also effective for health benefits. T2DM patients who walked more than 8000 steps/day were predicted to have healthier days, as they had lower HbA1c values compared to those with less than 4000 steps/day. Furthermore, a meta-analysis study that involved a total of 34,863 T1DM patients [51] demonstrated that diabetic patients with reduced PA presented low cardiorespiratory fitness and increased HbA1c concentration.

The practice of exercise and PA is able to promote important health benefits for T1DM patients. IDF also recommends the inclusion of muscle and bone strengthening activities at least three days a week and highlights their importance for maintaining plasma glucose close to recommended values as well as HbA1c at satisfactory concentrations. Similarly, as recommended by [14], small “daily doses” of PA associated with reductions in sedentary behavior, such as a decrease in sitting time, are capable of modestly attenuating post-prandial plasma glucose and insulin concentrations, particularly in individuals with insulin resistance and a high BMI.

In general, PA has great potential for the maintenance of glycemic homeostasis and health-related quality of life in DM individuals [52] and to mitigate its adverse effects on the genesis of microvascular complications [41,50,51,53].

Furthermore, studies corroborate that regular PA improves the function of b cells, insulin sensitivity, vascular function, and intestinal microbiota, which changes lead to the improvement of the health of T2DM patients at satisfactory levels, contributing to the improvement of their quality of life [54,55].

Therefore, according to the data presented so far, it appears that this evidence is in line with the main world recommendations for diabetes care of the American Diabetes Association (2023), ACMS (2022), the European Society of Cardiology (ESC), and the European Association for the Study of Diabetes (EASD) (2019) [13,14,15],which emphasize that for the control and prevention of DM (T1DM and T2DM), at least 150 min per week of moderate-intensity aerobic PA or 75 min per week of vigorous-intensity aerobic activity, or an equivalent combination of both, is required.

### 4.2. Level of Physical Activity and Its Relationship with Maintenance and Reduction of Body Weight and BMI in DM Patients during the COVID-19 Pandemic

The relationship between body weight and body mass index (BMI) for maintaining adequate blood glucose and HbA1c levels in DM patients (T1DM and T2DM) is an important factor that should be evaluated, especially in the context of COVID-19.

It is important to emphasize that the adipose tissue is an endocrine organ related to the increase/maintenance of body weight and BMI. The best-known fat pads are white and brown, which participate in the production and release of cytokines and act to maintain energy homeostasis. In addition to these, there is also the beige fat pad [56,57,58].

The following are among the main characteristics of beige adipose tissue: increase in mitochondrial number, increase in lipolytic activity and energy expenditure, increase in the expression of thermogenic genes, increase in vascularization and territorial blood flow, and promotion of hormone secretion [56,57,58]. In addition, physical exercise, through myokines, acts in a paracrine and autocrine manner in maintaining lipolytic activity and temperature increase [57,58,59].

In this way, brown adipose tissue provides beneficial effects on insulin sensitivity and homeostasis in lipid metabolism [38]. However, the increase in white adipose tissue is highly associated with cardiometabolic risks.

Some authors suggest that physical inactivity associated with sedentary behavior may contribute to the increase in white adipose tissue [12,13,14], which can result in increased body weight, especially the accumulation of adipose tissue in the abdominal region. Thus, it may result in metabolic alterations that culminate in low-grade systemic inflammation. The accumulation of white adipose tissue can also result in chronic inflammation, which can contribute to the development of metabolic diseases and DM imbalance [38,57].

In the opposite and beneficial way, PA and the regular practice of exercise play a protective role against chronic inflammation and reduce abdominal fat. These effects are important, especially in patients diagnosed with cardiometabolic diseases such as DM, hypertension, and dyslipidemia [58,60,61].

In this context, predominantly aerobic physical exercises have beneficial effects on the amount of circulating lipids and lipoproteins, mainly on the increase in HDL cholesterol, decrease in VLDL cholesterol, and triglycerides, which are directly related to the control of body weight, BMI, and worsening of preexisting diseases [38]. In addition, resistance exercises (anaerobic predominance) are fundamental for the improvement of HbA1c reduction parameters, anti-inflammatory therapy, metabolism improvement, and decrease of C-reactive protein levels in T2DM, in addition to the reduction of HbA1c, insulin dose/day, improvement of the cardio capacity (when combined), increase in strength, and improvement of the lipid profile in T1DM [62,63,64,65,66].

In this context, the literature corroborates that maintaining and/or increasing body weight at levels considered overweight and obesity (BMI > 26 and >30, respectively) is strongly associated with visceral adiposity, glucose intolerance, hypertension, dyslipidemia, endothelial dysfunction, and elevated levels of inflammatory markers [67]. According to Gal et al. (2022) [68], higher levels of PA, normal BMI, and sports practice are associated with increased time in range. Thus, in this study, our data suggested that T1DM patients may benefit from a high level of PA without fear of hypoglycemia.

Taking into account the importance of body weight reduction for BMI control, it is evident that weight reduction can contribute to important effects such as a decrease in HbA1c, blood lipids, and blood pressure. If necessary, especially for T2DM individuals, performing physical exercises of moderate volume at high intensity with expenditures of ~500 kcal/exercise session and a frequency of 4 to 5 days a week is recommended [14].

However, it should be emphasized that even if there is no body weight reduction, regular PA contributes to reducing visceral and subcutaneous fat, preventing weight gain [14].

In studies included in this review, Al Agha et al. (2021) [31] identified a significant increase in BMI and body weight during confinement in T1DM patients. Similarly, in the study by Ruissen et al., (2021) [33], 40.9% of T1DM participants reported weight gain and 45.7% reported less frequency of exercise compared to the period prior to restrictions, which was associated with body weight gain.

According to studies and the main international guidelines on DM, body weight reduction is an important strategy for controlling T2DM, and it is recommended that overweight and obesity be avoided since they act as causes of imbalance in important markers such as plasma glucose and HbA1c [12,13,14]. Similarly, according to K. Si et al. (2022) [69], losing weight is considered a primary control and effective strategy for prevention and management related to T2DM.

Corroborating these data, Zhao et al. (2022) [53] highlight that about 90% of T2DM individuals are overweight or obese, and obesity is strongly related to T2DM, mainly due to its association with insulin resistance. In a previously published review involving 17 studies, 14 observational studies, and 3 randomized studies with T2DM individuals, Strelitz et al. (2022) [39] concluded that weight gain is associated with increased risks of cardiovascular diseases (CVD) and mortality.

Therefore, body weight control in individuals with normal BMI, as well as reduction of adipose tissue in overweight and obese patients, becomes essential for health care and control of adequate blood glucose and HbA1c levels, especially in T1DM and T2DM patients. This objective has proven to be increasingly complex, given the scenario of COVID-19 control measures.

### 4.3. Restrictive COVID-19 Measures and Glycemic and HbA1c

The restrictions imposed with the aim of containing the proliferation of SARS-CoV-2 contributed to the worsening of the clinical condition of DM patients (T2DM and T2DM). In a study included in this review with T2DM patients [32] stress levels related to isolation measures were associated with decreased levels of physical exercise, increased total caloric intake, and increased body weight.

A systematic review that included 22 studies showed that in most articles analyzed, DM patients (T1DM and/or T2DM) consumed more fruits, vegetables, and grains and showed decreased desire for fast food and alcoholic beverages during the lockdown period [70]. Furthermore, some studies showed increased consumption of snacks and sweets, which resulted in disturbances in glycemic control and anthropometric values [70].

In this context, a review that included 28 studies with 5048 T1DM patients showed that the COVID-19 pandemic was associated with small improvements in glycemic control and without sufficient evidence regarding adjustments in HbA1c concentration [71]. It is noteworthy that this sample population had access to technology and lived in countries with high purchasing power.

There is no consensus in the literature on the effects of adopting isolation measures imposed by the global pandemic. Some authors suggest that the implementation of restrictions during the COVID-19 pandemic did not worsen glycemic control in T2DM patients. In the same study, triglyceride concentrations were high [72].

In another study, isolation measures to contain COVID-19 did not negatively impact glucose control, even showing a decline in PA among T1DM and T2DM individuals [72].

On the other hand, a robust study involving the participation of 16,895 T2DM patients evaluated the effects of COVID-19 lockdown on glycemic control and lipid profiles. In that study, lockdown to contain the spread of COVID-19 resulted in a significant increase in HbA1c, fasting glucose, and BMI levels in T2DM patients [73]. However, in this study, social isolation did not change the lipid parameters of this population.

Despite the variability in the outcomes of evaluated parameters, it is suggested that diabetic patients with pre-existing diseases such as obesity, DM, and hypertension may progress to more severe COVID-19 cases.

### 4.4. COVID-19 and Risks for Patients Diagnosed with Diabetes

One of the key factors when verifying glycemic control actions, especially those related to adequate levels of PA in T1DM and T2DM patients and taking into account the context of COVID-19, is that the imbalance of metabolic parameters (blood glucose, HbA1c, insulin, triglycerides, LDL, etc.) is directly related to the severity of COVID-19 if these individuals are infected by the SARS-CoV-2 virus [2,7,8].

SARS-CoV-2 infection, mainly in T1DM and T2DM individuals, triggers a cascade of deleterious health effects: COVID-19, DM decompensation, cytokine storm, and the worsening of previous cardiometabolic diseases [74].

This condition, aggravated by infection with COVID-19, causes immune dysregulation, severe inflammation, microvascular dysfunction, and thrombosis [75,76]. It has been identified that hyperglycemia, even in the short term, reduces innate immune function, and as a consequence of a malfunctioning innate immune response, DM patients (T1DM and/or T2DM) also have impaired adaptive immune function [75], which can cause health risks and contribute to the aggravation of COVID-19 [75].

Thus, patients with diabetes per se are more susceptible to developing cardiometabolic disorders and potentiate comorbidities that include insulin resistance, hypertension, and dyslipidemia, and the worsening of these parameters can contribute to the worse outcomes of the clinical condition of COVID-19 [76,77,78,79].

In view of the above, it is observed that numerous mechanisms have been proposed to justify the high vulnerability of DM patients to SARS-CoV-2, such as greater cell binding affinity, high virus entry, reduced viral removal, decreased T-cell function, high vulnerability to hyper-inflammation, cytokine storm, and the occurrence of CVD [74].

In short, whether it is potentiated by obesity and/or T2DM, inflammation is caused by changes in innate and adaptive immunity, high levels of circulating pro-inflammatory cytokines, including TNF-α, MCP-1, and IL-6, elevation of prothrombotic factors, high viral entry, and decreased viral clearance [2,3,4,68]. Furthermore, due to dyslipidemia, there is an increase in triglycerides, free fatty acids, inflammatory cytokines, insulin resistance, and C-reactive protein (CRP), which increase the risk of severe COVID-19 cases and mortality [2,3,4,68]. Thus, when it comes to DM, some authors suggest that the prevalence of chronic non-communicable diseases (NCDs), mainly DM, has been the main cause of the development and worsening of the health status of patients diagnosed with COVID-19 [8,78].

Thus, several authors suggest that COVID-19 promotes deleterious metabolic adjustments in DM patients, with an aggravation in the maintenance of plasma glucose in these patients. It was also found that glucose values close to recommended values as a control during hospitalization were associated with a lower mortality rate. Unlike patients with hyperglycemia, who were more susceptible to a high mortality rate, these results suggest that T2DM individuals hospitalized for COVID-19 with decompensated glucose had a high risk of death compared to others [22].

From the perspective of multiple cardiometabolic diseases, patients with hypertension, DM, and obesity had a longer length of stay in the intensive care unit (ICU) and a poorer prognosis [78]. In this regard, it is evident that DM is of worrying importance since it is associated with a high risk of serious infections and is a major cause of mortality [8,9].

It should be highlighted that the risk of evolution with the need to stay in the ICU among DM patients is 14.2% higher than among patients without DM [10]. Therefore, DM (T1DM and T2DM), whether alone or in combination with other comorbidities, can contribute to the worsening of COVID-19 symptoms and to the increase in the number of deaths in the world population [9,12]. These findings suggest that T1DM and/or T2DM patients may be at high risk of serious infections and mortality in the context of COVID-19 [7,8].

### 4.5. Weaknesses Detected during Article Analysis and Suggestions for Future Studies

It is important to highlight that all articles included in this review significantly contributed to a more detailed understanding of the researched topic as well as to promoting new perceptions of the way to approach the research problem and outcomes in view of the COVID-19 pandemic and its relationships with DM patients, which are widely discussed in the health area with academic and scientific societies. Thus, there is no intention of discrediting the commitment and investment of the authors of any work included in this systematic review.

The main weaknesses observed in selected studies were related to the selection of participants and data collection. Included studies did not mention and/or use inter-rater and intra-rater blinding and also did not report the order of assessment, as recommended by the Newcastle-Ottawa Risk of Bias Scale for Cohort and Case-Control Studies [80]. Furthermore, these studies showed low representativeness of the exposed cohort, as shown by the number of individuals participating in the study, which was mentioned by the studies in their topic of weaknesses and limitations. In addition to the assessment of exposure, data collection for the evaluation of results was carried out based on written self-reports or by questionnaire via telephone communication, as explained by the context of restrictions [30,36].

The follow-up time sufficient for results to occur can be considered a weakness in view of variations in the time of studies since the time of restrictions imposed by each country where studies were carried out was followed.

Furthermore, it could not be determined whether one or more of these metabolic parameters, in isolation and/or in combination, may contribute to the severe health condition imposed by COVID-19.

### 4.6. Study Limitations

The current systematic review has relevant strengths regarding the role of physical activity as an important tool for controlling blood glucose and HbA1c in DM patients (T1DM and T2DM), reducing stress levels, controlling body weight and BMI, and improving quality of life.

It has also been demonstrated that adequate levels of physical activity are important for the control and improvement of DM markers. In addition, this study has a low risk of publication bias.

However, the low number of articles and criteria for more specific types of physical activity (type, intensity, frequency, and number of weekly sessions) were among the study’s limitations.

Nevertheless, future studies should individualize the type of diabetes; determine the type of insulin, dosage, and frequency of administration; detail the delimitation of the sample based on age and sex; and determine the type, frequency, and duration of physical activity and/or physical exercise, with emphasis on which are the best training strategies to ensure the effectiveness of the study outcome.

## 5. Conclusions

Maintaining minimum levels of physical activity as recommended by the main international guidelines is significant and beneficial for controlling parameters related to diabetes mellitus, especially in the context of restrictions imposed during the COVID-19 pandemic. Furthermore, higher levels of physical activity are able to promote health benefits such as improved body composition (reduction in body weight and BMI), reduction in blood glucose concentration, and HbA1c close to recommended limits, thus resulting in improved cardiovascular health and glycemic homeostasis.

The practice of physical exercise (with guided frequency, duration, and intensities) prescribed by a physical education professional, combined with adequate levels of PA and the adoption of healthy eating habits, is capable of providing better health and quality of life.

In addition, a decrease in sedentary behavior can also contribute to attenuating and/or reducing the adverse effects triggered by cardiometabolic diseases. Predominant aerobic and resistance exercises of moderate to vigorous intensity can have a significant effect on HbA1c and blood glucose in DM patients (T1DM and T2DM).

Therefore, as an adjuvant therapy for people diagnosed with DM (T1DM and/or T2DM), the practice of at least 30 min of moderate to vigorous aerobic activity daily is recommended, associated with the practice of vigorous activities for muscle and bone strengthening of at least three days a week to control the disease [12,13].

The practice of physical exercise and/or physical activity by diabetic individuals (T2DM and/or T2DM) is important for maintaining health, as it may reduce the possibility of developing health problems when contaminated by COVID-19.

## Figures and Tables

**Table 1:** Main characteristics of eligible studies.

Author, Year of Publication and Country of Origin	Al Agha et al., 2021 Saudi Arabia [31]	Assolani et al., 2020 Italy [34]	Dalmazi et al., 2020 Italy [35]	Ruissen et al., 2021 The Netherlands [33]	Tornese et al., 2020 Italy [37]	Minuto et al., 2021 Italy [36]	Munekawa et al., 2021 Japan [32]
Place and/or region	King Abdulaziz University Hospital (KAUH) in Jeddah	Unspecified	Policlinico S. Orsola-Malpighi” in Bologna	Leiden University Medical Center, in Leiden, Holanda	Diabetes Pediatric Unit of the Institute for Maternal and Child Health ‘‘Burlo Garofolo’’, in province of Trieste	G. Gaslini Hospital, Regional Diabetes Center IRCCS Istituto Giannina Gaslini, University of Genova	Department of Endocrinology and Metabolism, Kyoto Prefectural University of Medicine, Kyoto
Study Design	Descriptive study: cross-sectional	Cohort study: observational	Cohort study: observational	Cohort study: observational	Cohort study: retrospective	Cohort study: cross-sectional and retrospective	Cohort study: cross-sectional and retrospective
Sample size and sex	48 (♂) 102 (♀)	71 (♂) 83 (♀)	30 children (13 ♀) 24 adolescents (9 ♀) 76 adults (37 ♀)	252 (♂) 183(♀)	5 (♂) 8(♀)	107 (♂) 95(♀)	126 (♂) 77(♀)
Sample age	8–16 years	32–44 years	18–47 years	52–65 years	11–14 years	6–39 years	56–65 years
Mean age	12.4	44.8	8.8 (7.7–10.6) 15.6 (14.2–16.8) 45.0 (29.0–58.1)	56.3	14.2	18.3	67.4
Type of diabetes	T1D	T1D	T1D	T1D and T2D	T1D	T1D	T2D
Age at diagnosis (years)	T1D 8.23 ± 5.34	-	4.2 (2.3–6.5) 7.2 (5.1–9.5) 22.0 (14.3–30.8)	-	-	The median duration of the disease was 9 years.	-
Insulin administration	15.3%—twice a day 50.7%— three times a day 24.7% to 32.7%—> four times a day, during confinement. 9.3%—by insulin pump.	-	Mean insulin dose (U/kg/day) 0.8 (0.6–0.8) 0.8 (0.7–1) 0.5 (0.4–0.6)	-	Total daily dose (U/kg per day) 57 (42–67) (U/kg per day) 0.9 (0.8–1.1) Basal amount (%) 57 (49–63) Basal amount (%) 50 (37–53)	168 (83.2%) CSII 34 (16.8%) MDI	135—Did not use insulin 68—Used insulin (unspecified dosages)
BMI (average)	20.6 kg/m^2^	24.7 kg/m^2^	−0.2 (−0.5–0.4) 21.3 (19.8–23.1) 24.7 (22.1–26.8)	27.5 kg/m^2^	-	-	28.4 kg/m^2^
Outcome	Change in lifestyle and eating habits during confinement ↑ predisposition to uncontrolled blood glucose	↓ PA ↓ number of steps ↓ PE ↑ Mean blood glucose (during 7 days of continuous CGM monitoring)	-	= HbA1c	= HCL in adolescents with T1D ↑ HCL associated to PA practice during pandemic period	↑ TIR in patients aged 14 years ↓ AF during confinement	↓ PE ↑ Total diet ↑ HCFI ↑ body weight ↑ HbA1c (men only) ↑ Stress
Impact	Physical activity patterns and diabetes control habits	Need for recommendations for exercise during periods of confinement	-	↑ Stress ↑ Anxiety ↑ weight gain ↓ PE ↓ Glycemic control without being associated with its deterioration	Regular PA and routine exercise in the home environment is an essential strategy for healthy living during the COVID-19 crisis, especially for young individuals with T1D	↑ Lifestyle ↓ Mean blood glucose (during 7 days of continuous CGM monitoring) in young patients with T1D A healthier lifestyle and Improved glycemic control	↑ stress levels caused by isolation ↓ PE ↑ total caloric intake ↑ body weight in T2D patients

Abbreviations: ♂: Male; ♀: Female; T1D: Type 1 diabetes; T2D: Type 2 diabetes; -: Unspecified referent information; U/kg/day: Insulin dose/unit per body weight per day; CSII: Continuous subcutaneous insulin infusion; MDI: Multiple daily injection; BMI: Body mass index; kg/m^2^: Kilogram per square meter; ↑: Increase; ↓: Decrease; PA: Physical activity; PE: Physical Exercise; CGM: Continuous glucose monitoring; TIR: Time in range; HCFI: High calorie food intake; HbA1c: Glycated hemoglobin; =: No changes in evaluated parameters; HCL: Hybrid closed loop.

**Table 2:** Main individual results of the observed variables.

Study	Group	Glucose (mg/dL)—Before	Glucose (mg/dL)— During	Glucose—Before vs. After (*p* Value)	HbA1c (mmol/mol; %)—Before	HbA1c (mmol/mol; %)—After	HbA1c— Before vs. After (*p* Value)	BMI (Mean)	Levels of Physical Activity (PA)—Before	Levels of Physical Activity (PA)—After	PA Before vs. After (*p* Value)
Al Agha et al., 2021 [31]	T1D	182.2 ± 76.6	200.45 ± 79.97	*p* < 0.007	7.45 ± 1.67%	7.40 ± 1.54	0.765	20.6 kg/m^2^	40.5% <30 min 28.0% <60 min 27.4% Inactive	↓ 66.1% ↑ 19.0% Did not affect 14.9%	*p* < 0.001
Assolani et al., 2020 [34]	T1D	142.1 ± 25.4	150.8 ± 29.4 mg/dL	*p* < 0.001	52.0 ± 0.9 6.9 ± 0.9%	-	-	24.7 kg/m^2^	Minutes: 66 ± 42	Minutes: 38 ± 31	*p* < 0.001
Dalmazi et al., 2020 [35]	T1D	-	-	-	57 (51–62) 51 (46–57) 56 (49–64)	-	-	−0.2 (Cr) 21.3 (Ad) 24.7 (Id)	IPAQ 1440 1018 1680	-	-
Ruissen et al., 2021 [33]	T1D	-	-	No impact	T1D 8%—12% T2D 8%—12%	-	No impact	27.5 kg/m^2^	-	>45.7%	*p* < 0.001
Tornese et al., 2020 [37]	T1D	155 (152–168)	152–168	-	-	-	-	-	76% regular PA	-	-
Minuto et al., 2021 [36]	T1D and T2D	176.16 ± 29.87	170.18 ± 30.14	*p* < 0.001	7.76 ± 1.04	7.56 ± 1.05	*p* < 0.001	-	Sport (h/week) 4.64 ± 4.24	Sport (h/week) 2.46 ± 3.22	*p* < 0.001
Munekawa et al., 2021 [32]	T2D	-	-	-	7.5 (±1.0)%	7.5 (1.0)% a 7.6 (1.1)%	*p* = 0.001	28.4 kg/m^2^	133 without habit 70 with habit	Lower PA > 50%	*p* < 0.001

Abbreviations: mg/dL: Milligrams per deciliter; HbA1c: Glycated hemoglobin; mmol/mol; %: percentage of millimoles per liter; BMI: Body mass index; kg/m^2^: Kilogram per square meter; PA: Physical activity; T1D: Type 1 diabetes; T2D: Type 2 diabetes; -: Unspecified referent information; ↓: Decrease; ↑ Increase; IPAQ: International physical activity questionnaire; h/week: Hours per week; before pandemic period: Before; during pandemic period: During; after pandemic period: After.

**Table 3:** Assessment of the methodological quality.

Author (year)	1	2	3	4	5	6	7	8	9	10	11	12	13	Quality
Al Agha et al., 2021 [31]	y	y	y	n	n	n	y	y	abs	n	abs	y	y	7
Munekawa et al., 2021 [32]	y	y	y	n	n	n	y	n	abs	y	abs	y	y	8
Ruissen et al., 2021 [33]	y	y	y	n	n	n	y	n	abs	y	abs	y	y	7
Assolani et al., 2020 [34]	y	y	y	n	n	n	y	n	abs	y	abs	y	y	7
Dalmazi et al., 2020 [35]	y	y	y	n	n	n	y	n	abs	y	abs	y	y	7
Minuto et al., 2021 [36]	y	y	y	n	n	n	y	y	abs	y	abs	y	y	8
Tornese et al., 2020 [37]	y	y	y	n	n	n	y	y	abs	y	abs	y	y	8

Abbreviations: 1—Sample adequacy; 2—adequacy description of evaluators; 3—explanation of the reference standard; 4—Inter-rater blinding; 5—Intra-rater blinding; 6—Variation of the evaluation order; 7—Period of time between the evaluated test and the reference standard; 8—Period between repeated measurements; 9—Independence from the reference standard of the evaluated test; 10—Adequacy of description of the evaluated test procedure; 11—Adequacy of description of the reference standard procedure; 12—Explanation about sample loss; 13—Appropriate statistical methods; y: component suitable; n: component not suitable; abs: absent and/or not mentioned.

## Data Availability

All original materials prepared for the study are included in the article/complementary material, and other questions can be forwarded to the corresponding author(s).
